# Coating-Dependent Neurotoxicity of Silver Nanoparticles—An In Vivo Study on Hippocampal Oxidative Stress and Neurosteroids

**DOI:** 10.3390/ijms23031365

**Published:** 2022-01-25

**Authors:** Katarzyna Dziendzikowska, Jacek Wilczak, Wojciech Grodzicki, Joanna Gromadzka-Ostrowska, Małgorzata Węsierska, Marcin Kruszewski

**Affiliations:** 1 Department of Dietetics, Institute of Human Nutrition Sciences, Warsaw University of Life Sciences—SGGW, Nowoursynowska 159C, 02-776 Warsaw, Poland; wojciech_grodzicki@sgge.edu.pl (W.G.); joanna_gromadzka_ostrowska@sggw.edu.pl (J.G.-O.); 2 Department of Physiological Sciences, Institute of Veterinary Medicine, Warsaw University of Life Sciences, Nowoursynowska 159, 02-776 Warsaw, Poland; jacek_wilczak@sggw.edu.pl; 3 Laboratory of Neuropsychology, Nencki Institute of Experimental Biology, Polish Academy of Sciences, 3 Pasteur Street, 02-093 Warsaw, Poland; m.wesierska@nencki.edu.pl; 4 Centre for Radiobiology and Biological Dosimetry, Institute of Nuclear Chemistry and Technology, Dorodna 16, 03-195 Warsaw, Poland; m.kruszewski@ichtj.waw.pl; 5 Department of Molecular Biology and Translational Research, Institute of Rural Health, Jaczewskiego 2, 20-090 Lublin, Poland

**Keywords:** silver nanoparticles, nanoparticle coating, hippocampus, neurosteroids, oxidative stress, antioxidative defense

## Abstract

Silver nanoparticles (AgNPs) are one of the most widely used nanomaterials. The level of exposure to nanosilver is constantly raising, and a growing body of research highlights that it is harmful to the health, especially the nervous system, of humans. The potential pathways through which nanosilver affects neurons include the release of silver ions and the associated induction of oxidative stress. To better understand the mechanisms underlying the neurotoxicity of nanosilver, in this study we exposed male Wistar rats to 0.5 mg/kg body weight of AgNPs coated with bovine serum albumin (BSA), polyethylene glycol (PEG), or citrate, or to AgNO_3_ as a source of silver ions for 28 days and assessed the expression of antioxidant defense markers in the hippocampus of the exposed animals after 1 week of spatial memory training. We also evaluated the influence of AgNPs coating on neurosteroidogenesis in the rat hippocampus. The results showed that AgNPs disrupted the antioxidant system in the hippocampus and induced oxidative stress in a coating-dependent manner, which could potentially be responsible for neurodegeneration and cognitive disorders. The analysis of the influence of AgNPs on neurosteroids also indicated coating-dependent modulation of steroid levels with a significant decrease in the concentrations of progesterone and 17α-progesterone in AgNPs(BSA), AgNPs(PEG), and Ag^+^ groups. Furthermore, exposure to AgNPs or Ag^+^ resulted in the downregulation of selected genes involved in antioxidant defense (*Cat*), neurosteroid synthesis (*Star*, *Hsd3b3*, *Hsd17b1*, and *Hsd17b10*), and steroid metabolism (*Ar*, *Er1*, and *Er2*). In conclusion, depending on the coating material used for their stabilization, AgNPs induced oxidative stress and modulated the concentrations of steroids as well as the expression of genes involved in steroid synthesis and metabolism.

## 1. Introduction

The beginning of the 21st century saw rapid development of nanotechnology in almost every branch of modern science and technology, facilitating its use in a wide range of commercially available products [1]. The nanomaterial most commonly found in consumer products is nanosilver according to the Nanodatabase [2]. Due to their desirable physicochemical properties, antimicrobial effect, and ease of synthesis, silver nanoparticles (AgNPs) have been successfully applied in hygiene products, cosmetics, textiles, toys, food containers, and dietary supplements [1,3]. However, the widespread use of AgNPs has increased the level of human exposure to these nanomaterials, mainly by inhalation, dermal contact, and ingestion [4]. Numerous studies in recent years have shown that AgNPs elicit toxic effects in various tissues, including the nervous system [5,6,7,8], which raises legitimate concerns about their possible impact on human health.

As proven by in vitro experiments, nanosilver is capable of penetrating the cells of organisms and can be detected in high amounts in cytosol, endosomes, and lysosomes [9]. In human cell lines, exposure to AgNPs results in cytotoxicity and DNA damage, while in animal models nanosilver can affect multiple organs, cause reproductive system dysfunction, and alter brain functions [10,11,12,13]. Given the crucial role of the nervous system in the regulation of key vital functions, the neurotoxic potential of AgNPs has received special attention among researchers. In fact, nanoparticulate silver can cross the blood–brain barrier and accumulate in the brain, with an exceptionally slow excretion rate [14]. The presence of AgNPs in the nervous tissue has been linked to several adverse effects, such as decreased learning capacity and worsened social activity, indicating the disruption of hippocampal functions [15,16].

One of the principal mechanisms through which nanomaterials exert toxicity is oxidative stress [17]. Nanosilver increases the formation of reactive oxygen species (ROS) and thus contributes to the depletion of antioxidant defense capacity, alterations in gene expression, inflammation, impairment of mitochondrial functions, DNA damage, and eventually, neuronal death [11,13,18]. In addition, nanosilver can promote detrimental processes, such as the formation of amyloid-beta plaques, which is observed in Alzheimer’s disease, and may be a risk factor for neurodegeneration [18,19].

The toxicity of AgNPs is determined by several factors, such as the “Trojan horse effect” which involves the intracellular release of reactive silver ions. However, nanoparticles may themselves exhibit toxic potential, which is at least partially influenced by the type of surface coating used for their stabilization [11]. The functionalization of AgNPs has an impact on their bioavailability and interactions with plasma proteins. Under physiological conditions, plasma proteins adsorb on the surface of nanoparticles, forming a protein corona. The chemical composition of coating that enables the adsorption of different proteins determines the interaction of AgNPs with cells, and consequently, the fate of these nanoparticles in the exposed organism [1,20]. However, the mechanisms by which different surface modifications influence the neurotoxicity of nanosilver remain largely unknown.

Considering the extensive application of nanosilver in consumer products and increased human exposure, in this study we investigated the neurotoxic impact of AgNPs by analyzing the effects of differently coated AgNPs on the antioxidant parameters, oxidative stress, and neurosteroid metabolism in the hippocampus of orally exposed rats after 1 week of memory training.

## 2. Results

### 2.1. Neurosteroid Level in the Hippocampus

**Figure 1:**
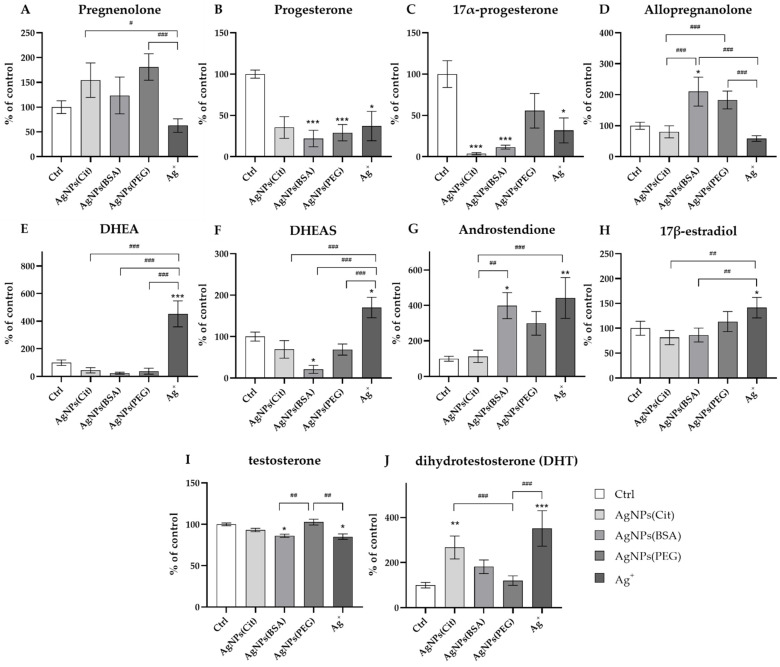
Neurosteroid levels in the hippocampus of rats exposed to AgNPs with different types of coating (citrate, bovine serum albumin (BSA) and polyethylene glycol (PEG)) or Ag^+^: (**A**)—pregnenolone; (**B**)—progesterone; (**C**)—17α-progesterone; (**D**)—allopregnanolone; (**E**)—dehydroepiandrosterone (DHEA); (**F**)—dehydroepiandrosterone sulfate (DHEAS); (**G**)—androstenedione; (**H**)—17β-estradiol; (**I**)—testosterone; (**J**)—dihydrotestosterone (DHT). Data are expressed as mean ± SEM. *^,^ **^,^ *** Significantly different from the control group (* *p* < 0.05, ** *p* < 0.01, *** *p* < 0.001) (Tukey post hoc test). ^#, ##, ###^ Significant differences between the groups exposed to AgNPs or Ag^+^ (^#^ *p* < 0.05, ^##^ *p* < 0.01, ^###^ *p* < 0.001) (Tukey post hoc test).

The results of ANOVA revealed that the concentrations of all neurosteroids in the rat hippocampus were significantly influenced by AgNPs depending on the type of coating used on the nanoparticles. The hippocampal concentration of pregnenolone did not significantly differ between the groups exposed to AgNPs with different types of coating, the Ag^+^ group, and the control group (Ctrl) (Figure 1A). However, the level of pregnenolone was found to be significantly lower in the rats from the Ag^+^ group compared to AgNPs coated with polyethylene glycol (PEG) and citrate (Cit) groups (ANOVA: *p* = 0.003; post hoc: *p* < 0.001 and *p* < 0.05, respectively).

The hippocampal concentration of progesterone was significantly decreased in AgNPs coated with bovine serum albumin (BSA), AgNPs(PEG), and Ag^+^ groups in comparison to the control group (ANOVA: *p* = 0.001; post hoc: *p* < 0.001, *p* < 0.001, and *p* < 0.05, respectively) (Figure 1B). A similar relationship was observed in the case of 17α-progesterone, the hippocampal concentration of which was found to be lower in AgNPs(Cit), AgNPs(BSA), and Ag^+^ groups in comparison to the control group (ANOVA: *p* = 0.001; post hoc: *p* < 0.001, *p* < 0.001, and *p* < 0.05, respectively) (Figure 1C). On the other hand, the hippocampal concentration of allopregnanolone was the highest in the rats from the AgNPs(BSA) group and significantly higher compared to the control group (ANOVA: *p* = 0.001; *post hoc*: *p* < 0.05). Additionally, the level of allopregnanolone in AgNPs(BSA) and AgNPs(PEG) groups was observed to be significantly increased compared to the rats in AgNPs(Cit) and Ag^+^ groups (post hoc: *p* < 0.001 for all comparisons) (Figure 1D).

The hippocampal concentrations of two pregnenolone metabolites, dehydroepiandrosterone (DHEA) and dehydroepiandrosterone sulfate (DHEAS), which are formed in different biotransformation pathways, were the highest in the Ag^+^ group (ANOVA: *p* = 0.001 for both cases). The levels of these two metabolites found in the Ag^+^ group significantly differed from those observed in the control group (post hoc: *p* < 0.001 and *p* < 0.05, respectively) and in all AgNPs-exposed animals (post hoc: *p* < 0.001 for all comparisons) (Figure 1E (DHEA) and Figure 1F (DHEAS)).

The concentrations of two metabolites of progesterone—androstenedione and 17β-estradiol—were also found to be the highest in the rats from the Ag^+^ group (ANOVA: *p* = 0.001 for both steroids). The hippocampal levels of both androstenedione and 17β-estradiol in this group significantly differed from the concentration noted in the control group (post hoc: *p* < 0.01 and *p* < 0.05, respectively). Moreover, androstenedione concentration in the rats exposed to AgNPs(BSA) was significantly higher than that in the control animals (post hoc: *p* < 0.05) (Figure 1G (androstenedione) and Figure 1H (17β-estradiol)). Additionally, the level of this neurosteroid was significantly higher in Ag^+^ and AgNPs(BSA) groups in comparison to the AgNPs(Cit) group (post hoc: *p* < 0.001 and *p* < 0.05, respectively). The concentration of 17β-estradiol was also higher in Ag^+^ rats than in AgNPs(BSA) and AgNPs(Cit) groups (post hoc: *p* < 0.01 for both cases).

In addition, the concentration of two neurosteroids belonging to the androgens group—testosterone and dihydrotestosterone (DHT)—differed depending on the type of nanosilver coating (ANOVA: *p* = 0.001 for both androgens). A lower hippocampal concentration of testosterone was observed in the Ag^+^ group compared to AgNPs(BSA) and AgNPs(Cit) animals (post hoc: *p* < 0.01 for both comparisons) (Figure 1I). Interestingly, the hippocampal level of DHT was higher in the rats from Ag^+^ and AgNPs(Cit) groups compared to that in the animals from the control group (post hoc: *p* < 0.001 and *p* < 0.01, respectively) as well as from the AgNPs(PEG) group (post hoc: *p* < 0.001 for both comparisons) (Figure 1J).

### 2.2. Antioxidant Potential and Oxidative Stress in the Hippocampus

**Figure 2:**
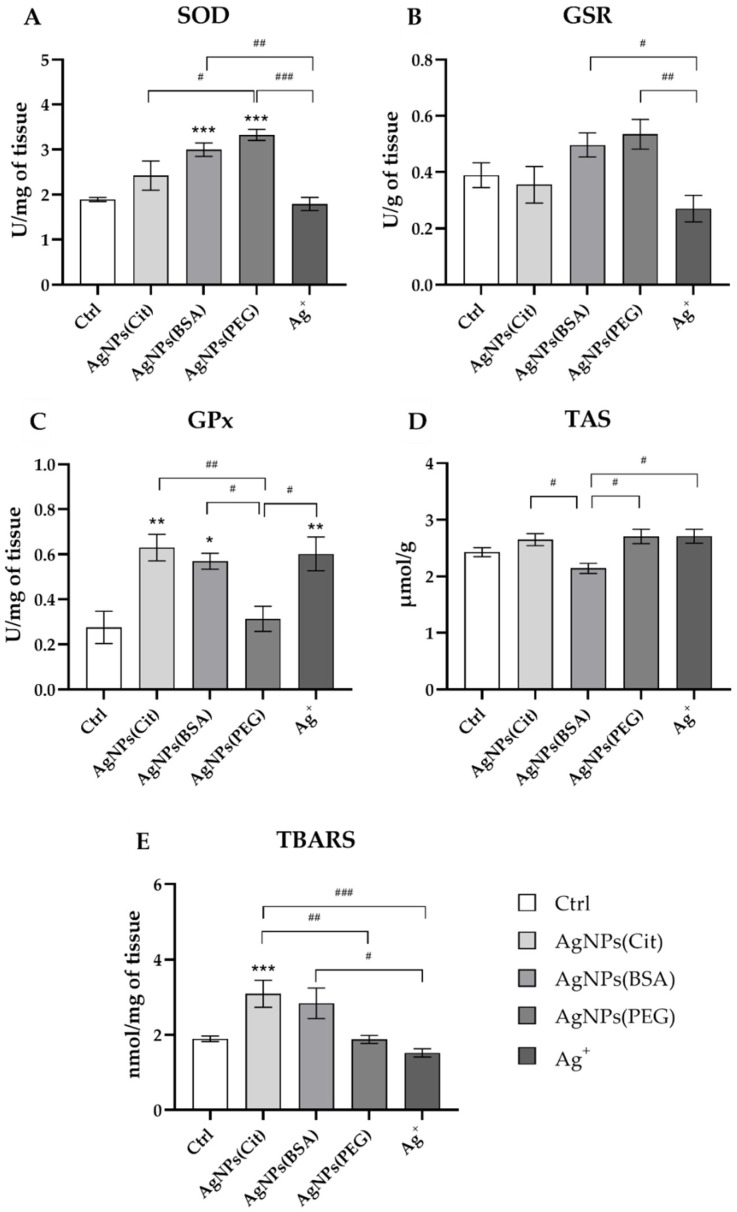
Antioxidant potential and oxidative stress in the hippocampus of rats exposed to AgNPs with different types of coating (citrate, bovine serum albumin (BSA), and polyethylene glycol (PEG)) or Ag^+^: (**A**)—superoxide dismutase (SOD); (**B**)—glutathione reductase (GSR); (**C**)—glutathione peroxidase (GPx); (**D**)—total antioxidant status (TAS); (**E**)—thiobarbituric acid-reactive substances (TBARS). Data are expressed as mean ± SEM. *^,^ **^,^ *** Significantly different from the control group (* *p* < 0.05, ** *p* < 0.01, *** *p* < 0.001) (Tukey post hoc test). ^#, ##, ###^ Significant differences between the groups exposed to AgNPs or Ag^+^ (^#^ *p* < 0.05, ^##^ *p* < 0.01, ^###^ *p* < 0.001) (Tukey post hoc test).

The statistical analysis revealed that the hippocampal activity of antioxidative enzymes was significantly influenced by AgNPs depending on the type of coating used. ANOVA indicated that different types of nanosilver coating showed different effects on the superoxide dismutase (SOD) activity in the hippocampus (ANOVA: *p* = 0.001) (Figure 2A). The post hoc test revealed statistically higher SOD activity in AgNPs(BSA) and AgNPs(PEG) groups than the control group (post hoc: *p* < 0.001 for both comparisons). Furthermore, significantly higher SOD activity was found in the AgNPs(BSA) group as compared to the Ag^+^ group (post hoc: *p* < 0.01). Similarly, the rats in the AgNPs(PEG) group exhibited an increased SOD activity in comparison to the animals in both Ag^+^ and AgNPs(Cit) groups (post hoc: *p* < 0.001 and *p* < 0.05, respectively).

The values of glutathione reductase (GSR) activity determined in the studied groups are presented in Figure 2B. The statistical analysis showed that GSR activity in rats was affected by silver administration (ANOVA: *p* = 0.008). Although no differences in GSR activity were found with regard to the control group, the post hoc test revealed that the GSR activity was significantly lower in the Ag^+^ group in comparison to the animals receiving either AgNPs(BSA) or AgNPs(PEG) (post hoc: *p* < 0.05 and *p* < 0.01, respectively).

Glutathione peroxidase (GPx) activity was also significantly influenced by treatment with silver (ANOVA: *p* = 0.001) (Figure 2C). As the post hoc test showed, GPx activity was significantly higher in the AgNPs(Cit), AgNPs(BSA), and Ag^+^ groups (post hoc: *p* < 0.01, *p* < 0.05, and *p* < 0.01, respectively) compared to that in the control group. In addition, the post hoc analysis revealed intergroup differences. Rats treated with AgNPs(PEG) displayed significantly lower GPx activity than the animals in the AgNPs(Cit) group, as well as those in AgNPs(BSA) and Ag^+^ groups (post hoc: *p* < 0.01, *p* < 0.05, and *p* < 0.05, respectively).

The total antioxidant status (TAS) values measured to assess the overall antioxidant status of the hippocampus in the studied groups are presented in Figure 2D. Although ANOVA revealed that the TAS level in the hippocampus was influenced by exposure to silver (ANOVA: *p* = 0.006), the post hoc test indicated that only animals treated with AgNPs(BSA) showed significantly different TAS values from the other Ag-exposed groups, that is, AgNPs(PEG), AgNPs(Cit), and Ag^+^ groups (post hoc: *p* < 0.05 for all comparisons).

The results of the analysis of lipid peroxidation, which are expressed as the concentration of thiobarbituric acid-reactive substances (TBARS), are presented in Figure 2E. The TBARS levels in the studied groups were also influenced by silver administration (ANOVA: *p* = 0.001). Statistically significantly higher TBARS concentration was found in the control group and the AgNPs(Cit) group (post hoc: *p* < 0.01). AgNPs(BSA) treatment resulted in a significantly higher TBARS concentration in comparison to Ag^+^ (post hoc: *p* < 0.05). Similarly, AgNPs(Cit) treatment resulted in much higher lipid peroxidation level than AgNPs(PEG) and Ag^+^ (post hoc: *p* < 0.01 and *p* < 0.001, respectively).

### 2.3. Gene Expression Analysis

The results of the analysis of antioxidant defense genes expression are shown in Table 1. Oral administration of 0.5 mg/kg b.w. of AgNPs with different types of coating or Ag^+^ for 28 days influenced the expression of the analyzed antioxidant defense/oxidative stress marker genes. According to ANOVA, catalase (*Cat*) expression was strongly influenced by silver (ANOVA: *p* = 0.006), with statistically significant downregulation observed in AgNPs (PEG)- and Ag^+^-treated animals (post hoc: *p* < 0.05 and *p* < 0.01, respectively). Similarly, glutathione reductase (*Gsr*) gene expression was downregulated in Ag^+^-treated group (ANOVA: *p* = 0.047, post hoc: *p* < 0.05). No statistically significant differences in the expression of superoxide dismutase 1 (*Sod1*), superoxide dismutase 2 (*Sod2*), glutathione peroxidase 1 (*Gpx1*), and heme oxygenase 1 (*Hmox1*) genes were found between the experimental groups or between the experimental groups and the control group.

**Figure 3:**
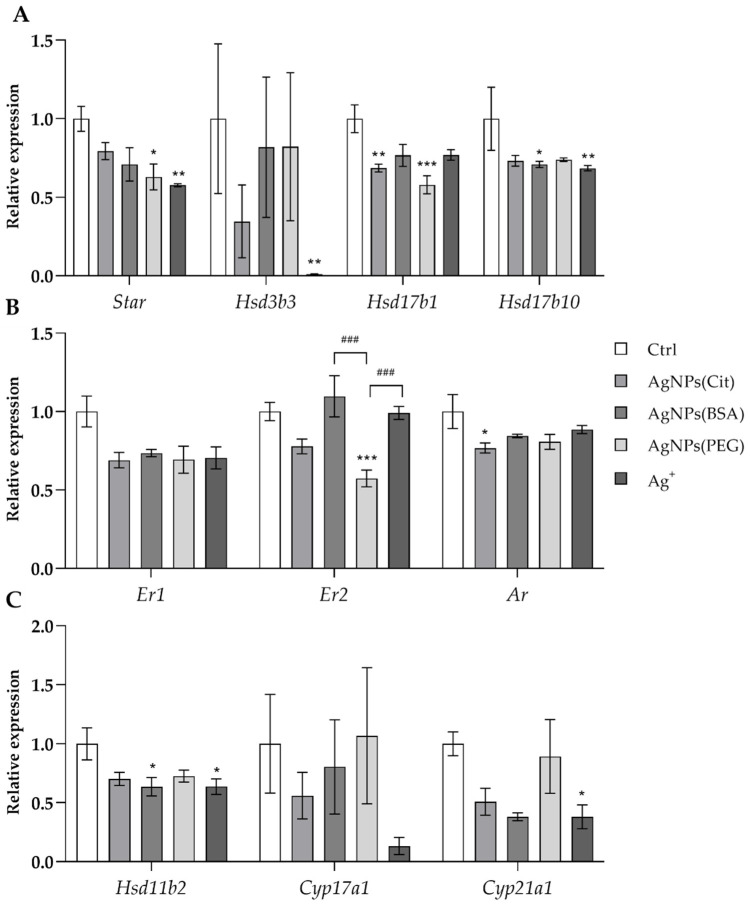
Relative expression of the genes involved in neurosteroid synthesis and metabolism vs. the reference phosphoglycerate kinase (*Pgk1*) gene in the hippocampus of rats exposed to AgNPs with different types of coating (citrate, bovine serum albumin (BSA), and polyethylene glycol (PEG)) or Ag^+^: PEG) or Ag^+^: (**A**)—steroidogenesis genes (*Star* (steroidogenic acute regulatory protein), *Hsd3b3* (hydroxysteroid (3β) dehydrogenase 3), *Hsd17b1* (hydroxysteroid (17β) dehydrogenase 1), *Hsd17b10* (hydroxysteroid (17β) dehydrogenase 10)); (**B**)—steroid metabolism genes (*Er1* (estrogen receptor 1), *Er2* (estrogen receptor), *Ar* (androgen receptor)); (**C**)—glucocorticosteroid synthesis genes (*Hsd11b2* (hydroxysteroid 11β-dehydrogenase 2), *Cyp17a1* (cytochrome P450, family 17, subfamily a, polypeptide 1), *Cyp21a1* (cytochrome P450, family 21, subfamily a, polypeptide 1)). Data are presented in arbitrary units as a ratio of the expression of the target gene to the expression of the reference gene (*Pgk1*) with the control group calculated as 1. All values are presented as mean ± SEM. *^,^ **^,^ *** Significantly different from the control group (* *p* < 0.05, ** *p* < 0.01, *** *p* < 0.001) (Tukey post hoc test). ^###^ Significant differences between the groups exposed to AgNPs or Ag^+^ (^###^ *p* < 0.001) (Tukey post hoc test).

The expression of selected genes involved in hippocampus neurosteroidogenesis was downregulated by AgNPs based on the different coating materials used for stabilization. The changes in expression were the most pronounced in the groups treated with AgNPs coated with PEG and citrate. A significant reduction in the expression of both genes involved in neurosteroidogenesis and neurosteroid metabolism was observed. ANOVA showed a decrease in the expression of genes encoding the key enzymes involved in steroidogenesis, including steroidogenic acute regulatory protein (*Star*), hydroxysteroid (3β) dehydrogenase 3 (*Hsd3b3*), hydroxysteroid (17β) dehydrogenase 1 (*Hsd17b1*), and hydroxysteroid (17β) dehydrogenase 10 (*Hsd17b10*) genes (ANOVA: *p* = 0.009, *p* = 0.019, *p* = 0.001, and *p* = 0.012, respectively) (Figure 3A, Table 2). 

The post hoc analysis showed that the expression of *Star* and *Hsd17b1* genes was lower in the AgNPs(PEG) group compared to the control group (*p* < 0.05 and *p* < 0.001, respectively). Similarly, the expression of *Star* and *Hsd3b3* genes was reduced in the Ag^+^ group compared to the control group (*p* < 0.01 and *p* < 0.05, respectively). The expression of *Hsd17b1* gene was lower in the AgNPs(Cit) group than in the control group (*p* < 0.01). Furthermore, downregulation of *Hsd17b10* expression was observed in AgNPs(BSA) and Ag^+^ groups in comparison to the control group (*p* < 0.05 and *p* < 0.01, respectively). No significant differences were noted in the expression of cytochrome P450, family 11, subfamily a, polypeptide 1 (*Cyp11a1*) and hydroxysteroid (17β) dehydrogenase 3 (*Hsd17b3*) genes (Figure 3A, Table 2).

In the case of the expression of genes involved in steroid metabolism, ANOVA revealed that the transcription levels of androgen receptor (*Ar*) and both estrogen receptor 1 and 2 (*Er1* and *Er2*) genes were reduced in the hippocampus of AgNPs-treated animals (ANOVA: *p* = 0.030, *p* = 0.001, and *p* = 0.039, respectively). *Ar* gene expression was reduced in the AgNPs(Cit) group compared to the control group (post hoc: *p* < 0.05), while *Er2* gene expression was lower in the AgNPs(PEG) compared to the control group (post hoc: *p* < 0.001) as well as AgNPs(BSA) and Ag^+^ groups (post hoc: *p* < 0.001 for both). No statistically significant differences were observed in the expression of *Srd5a1*, *Sult2a2*, and *Cyp19a1* (Figure 3B, Table 2).

Additionally, ANOVA revealed statistically significant downregulation of glucocorticosteroidogenesis genes, including hydroxysteroid 11β-dehydrogenase 2 (*Hsd11b2*), cytochrome P450, family 21, subfamily a, polypeptide 1 (*Cyp21a1*), and cytochrome P450, family 17, subfamily a, polypeptide 1 (*Cyp17a1*) (ANOVA: *p* = 0.029, *p* = 0.026, and *p* = 0.046, respectively). Post hoc analysis showed that the expression of *Hsd11b2* and *Cyp21a1* genes was lower in the Ag^+^ group, and the expression of *Hsd11b2* was lower in the AgNPs(BSA) group than in the control rats (*p* < 0.05 for all comparisons) (Figure 3C, Table 2).

### 2.4. Fisher’s Linear Discriminant Analysis

**Figure 4:**
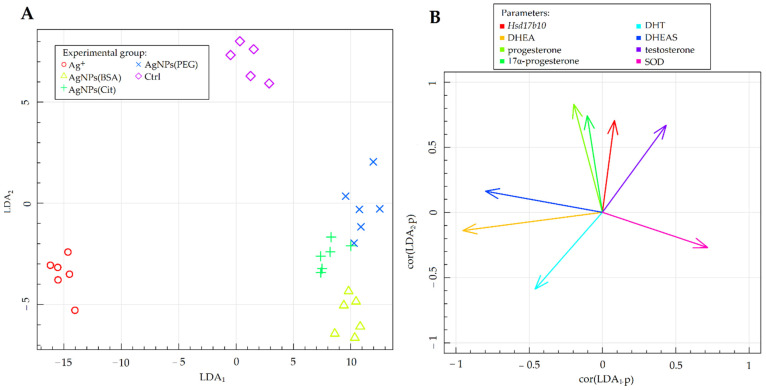
Fisher’s LDA: (**A**)—experimental data on the plane spanned by two of the most data-separating LDAs; (**B**)—parameters contributing the most to LDAs. *Hsd17b10*—gene expression of hydroxysteroid (17β-beta) dehydrogenase 10; DHEA—level of dehydroepiandrosterone; DHT—level of dihydrotestosterone; DHEAS—level of dehydroepiandrosterone sulfate; SOD—activity of superoxide dismutase.

Fisher’s LDA was carried out in order to summarize the results and obtain linear combinations (linear discriminants) of the studied parameters that allow the best distinction of the experimental groups. The results of Fisher’s LDA of the experimental data are illustrated in Figure 4. The vectors shown in Figure 4B clarified the correlations between the values of the relevant parameters and two of the most data-separating combinations (LDA_1_ and LDA_2_). Moreover, the vectors indicated the direction in which the related parameters specify the separation of the experimental groups presented in Figure 4A. The results of LDA help in understanding and organizing the variance analysis results in addition to confirming them.

The results of Fisher’s LDA revealed that the parameters that correlated the most between the LDA_1_ and LDA_2_ combinations, differentiating the studied experimental groups, were the following: (1) the levels of DHEA, DHEAS, progesterone, 17α-progesterone, testosterone, and DHT among the neurosteroids in the hippocampus; (2) the activity of SOD among the antioxidant defense enzymes; and (3) the expression of *Hsd17b10* among the genes involved in neurosteroid synthesis and metabolism (Figure 4B). The control group stood out from the remaining groups, irrespective of the chemical form of silver administered and the coating material used (Figure 4A). The experimental groups were differentiated based on the value of the LDA_2_ coefficient, which correlated the most with the levels of progesterone, 17α-progesterone, and testosterone, and the expression of *Hsd17b10* gene (positively correlated with LDA_1_). The Ag^+^ group was differentiated based on the value of the LDA_1_ coefficient, which correlated the most with the levels of DHEA, DHEAS, and DHT (positively correlated with LDA_1_), as well as the level of SOD (negatively correlated with LDA_1_). Importantly, this group was separated from the other groups that received nanoparticles with different types of coating, as reflected by its position relative to the horizontal axis showing the LDA_1_ coefficient (Figure 4A). The position of the Ag^+^ group on the graph is determined by the levels of neurosteroids and the parameters related to their synthesis and metabolism, including the levels of DHEA, DHEAS, and DHT, and the expression of *Hsd17b10* gene.

## 3. Discussion 

Due to the widespread use of AgNPs in the food industry and medicine, as well as the increasing environmental risk associated with exposure to AgNPs, researchers have been investigating the safety of nanomaterials use [9]. AgNPs are found in a wide range of household products used on a daily basis, such as biomedicine products, textiles, hygiene and personal care items, food storage supplies, and so on [21]. Additionally, AgNPs have been studied as potential drug delivery and radiosensitizing efficacies in gliomas as well as agents for diagnosing and treating neurodegenerative diseases [22]. This extensive usage of AgNPs and their proven ability to cross biological barriers, such as the blood–brain barrier, have raised concerns about their potential influence on brain functions. Results obtained by Recordati et al. showed high deposition of silver in the brain after oral exposure of environmentally relevant dose of citrate-coated AgNPs at the end of the administration as well as after 4 weeks of recovery that highlighted slow elimination of Ag from the brain structures [23]. Our previous study has also shown that oral administration of AgNPs at a relatively small concentration resulted in a time-dependent accumulation of silver in the brain, particularly in the hippocampus, and simultaneous deterioration of learning skills in rats [16,24]. Additionally, our recent studies demonstrated that the effect of AgNPs on cognitive functions is determined by the coating used for the stabilization of these nanoparticles. It was found that exposure to AgNPs coated with BSA and PEG, as well as AgNO_3_ as a source of Ag^+^, was associated with impairment of hippocampal-dependent cognitive functions [24]. Taking into account the results of our studies and the possible toxicity of AgNPs toward the central nervous system (CNS), in the present study we investigated the mechanisms underlying the observed impairment of cognition functions after oral administration of AgNPs at environmentally relevant concentrations.

Experimental evidence shows that the physicochemical properties of nanoparticles are one of the key factors determining their fate in the body [1,6]. Studies have indicated that besides particle size, synthesis methods, elemental composition, surface charge and area, aggregation, and dissolution in body fluids, surface coating also affects the tissue biodistribution and cellular uptake of nanoparticles as well as their reactivity and toxicity [6,25,26]. Of the mentioned factors, surface modification has the most significant influence on the immune system and is likely to determine the toxicity of nanoparticles [27,28]. For this reason, in the present study we attempted to elucidate the mechanisms responsible for the observed toxicity of AgNPs determined by the coating agent (BSA, PEG, citrate) used for their surface stabilization.

AgNPs used in the food packages limit the development of spoilage microorganisms by releasing Ag^+^ ions to generate ROS, which can destroy bacterial membrane and thus control the growth of microorganisms [29]. Although these functional properties of nanoparticles are advantageous for the food industry, they are undesirable as the nanoparticles come in contact with cells in the human body. Oxidative stress is proposed as the main mechanism behind nanoparticle-induced toxicity due to the release of Ag+ after infiltrating the cells and AgNPs intracellular degradation [26,30]. The brain is particularly susceptible to ROS because of its high oxygen consumption, weak antioxidative capability, and high content of peroxidation-prone unsaturated fatty acids [31]. Oxidative stress has been proven as an important pathogenesis factor in neurodegenerative diseases, such as Alzheimer’s and Parkinson’s disease. ROS may also induce neuronal cell death and brain inflammation. In addition, ROS modulate the functions of neuronal ion channels, contributing to long-term memory dysfunction [32].

Nanosilver has been known to induce oxidative stress in the CNS [33,34,35,36]. AgNPs increase the production of free radicals, provoke lipid and protein peroxidation, and disrupt mitochondrial functions [28]. Our previous studies have shown a time-dependent silver deposition in the brain and the induction of oxidative stress in the brain of animals injected with BSA-coated AgNPs [8,37] and in rats that inhaled diesel exhaust containing nanoparticles which included silver [38]. Though in the present study we did not directly assess the level of oxidative stress, some indirect evidence suggests that oxidative stress in the AgNPs-treated group was higher compared to the control group. The SOD activity was found to be significantly elevated in AgNPs(PEG) ond AgNPs(BSA) group compared to AgNPs(Cit), Ag^+^, and control groups. These results are in agreement with those of our previous study [8]. These findings are also in line with the study of Skalska et al. [34], in which no changes in SOD activity were observed in the brain of rats exposed to AgNPs(Cit), although the authors reported an increase in GPx activity. Our study confirmed this observation, as we found increased GPx activity in the groups exposed to AgNPs(Cit) or AgNPs(BSA). Similar findings were also reported by Dănilă et al. [39], who studied the effects of AgNPs(Cit) and AgNPs functionalized with polyphenols on the offspring of female rats treated with nanoparticles. The authors found that the SOD activity in the hippocampus and cerebellum did not change significantly in the Cit-AgNPs group, although it was lowered by exposure to AgNPs coated with plant extracts [39]. On the contrary, Yousef et al. [40] observed decreased SOD activity in the brain of rats treated with either AgNPs or AgNPs combined with Fe_2_O_3_ nanoparticles. In both groups of animals a significant decline of antioxidant enzymes activity (not only SOD but also CAT and GPx) was observed [40].

To elaborate the mechanisms behind the changes observed in the activity of antioxidant enzymes, we analyzed the impact of differently coated AgNPs on the expression of genes related to the antioxidant defense system (*Sod1*, *Sod2*, *Cat*, *Gpx1*, *Gsr*, *Hmox1*) as well as the level of TBARS and TAS in the hippocampus of the studied groups of rats. However, our results showed only downregulation of *Cat* in the AgNPs(BSA)-exposed group. This result is similar to that reported by Chen et al. [41], who observed no differences in the expression of *Sod1*, *Hmox1*, and *Gpx* in the brain tissue of mice treated intravenously with nanosilver. Dąbrowska-Bouta et al. [42] also observed no significant influence of AgNPs on the expression of *Sod1* gene in the rat brain cortex, although *Sod2* expression was elevated by nearly 30%. Conversely, Dayem et al. [35] found that the expression of *SOD2* and *CAT* was decreased in human neuroblastoma SH-SY5Y cells treated with AgNPs, while the expression of *GPX1* remained unchanged. Additionally, Davenport et al. [43] showed an impact of nanosilver on the expression of *Hmox1* in the hippocampi of intranasally exposed mice.

We found that AgNPs affected the overall antioxidant status of the hippocampus of exposed animals. While TAS was similar in the groups receiving PEG- or citrate-coated AgNPs or Ag^+^ as compared to the control group, it was found to be decreased significantly in the hippocampus of AgNPs(BSA)-treated animals. Analogous outcomes were reported by Yousef et al. [40], who found that the total antioxidant capacity of the brain was lowered by 22.4% in the group treated with AgNPs and by 35.8% in the group treated with AgNPs and Fe_2_O_3_. Similar results were also shown by Attia et al. [44], who also found reduced TAS levels in mice exposed to high doses of citrate-coated AgNPs. In addition, recently, Opris et al. [45] also found increased lipid peroxidation products and simultaneous severe ultrastructural changes in neurons and astrocytes in the hippocampus in rats orally exposed to AgNPs phytosynthesized with *Cornus mas* L. extract. This is in line with the results of our study, which also found elevated TBARS levels, but only in animals that received BSA-coated AgNPs. Taken together, the results suggest that AgNPs can affect the antioxidant defense system in the hippocampus. However, the exact effects of nanoparticles seem to vary depending on the specific form of silver used and the functionalization applied.

AgNPs-induced oxidative stress in the brain has been reported to promote astrocyte proliferation, probably as a response to protect nearby neurons. It is a kind of defense mechanism as astrocytes and other glial cells are the principal cells involved in brain response to injury and in the protection of the neurons against oxidative stress and metal toxicity [46]. Recordati et al. [23] showed dose-dependent glial cells activation after treatment with citrate-coated AgNPs, but not ionic Ag in mice orally exposed to AgNPs or silver acetate as a source of Ag ions. Additionally, they found swelling of astrocytic perivascular end-feet in both Ag-exposed mice. However, at higher concentrations AgNPs change astrocyte morphology and provoke caspase-dependent cytotoxicity, which eventually leads to cell death [36,46]. Simultaneously, neurons themselves can also be affected, as evidenced by inhibited neurite growth and reduction in the number of synapses [46]. Since astrocytes serve an important role in the supply of cholesterol, the primary substrate for neurosteroid synthesis in which both them and primary neurons are involved, these observations suggest that nanoparticulate silver could exert a detrimental effect on steroidogenesis in the brain [47,48]. Furthermore, in the intracellular space AgNPs-mediated ROS production has been shown to disrupt mitochondrial functions [6,13,18]. Steroidogenesis is one of the key functions of mitochondria [49]. Therefore, an important part of the present study was the analysis of whether AgNPs induced changes in the metabolism of neuroactive steroids, based on the assessment of steroid content and expression of genes involved in neurosteroid metabolism in the hippocampus. Neurosteroids are steroid hormones synthesized in the brain or peripheral neurons. They are either formed de novo from cholesterol or originate from peripheral tissues, such as endocrine glands including gonads and adrenal glands. The term “neuroactive steroids” refers to the steroids that act on the CNS [50]. Increased oxidative stress may also result from impaired steroid synthesis [51]. ROS inhibits steroidogenesis mainly by reducing the availability of the substrate for steroid synthesis by decreasing the expression of StAR protein in cells, as well as the activity of steroid biosynthetic pathway enzymes, including 3β-hydroxysteroid dehydrogenase and cytochrome P450scc associated with cholesterol desmolase [52]. On the other hand, AgNPs induce dysfunction of the mitochondria. The major mitochondrial ultrastructural changes noticed in animals treated with AgNPs suggested mitochondrial dysfunction as a response to stress conditions, consisting of swelling, altered cristae, and elongation [34,44,45]. Reported changes in mitochondrial functions are probably resulting from the interactions of Ag^+^ ions with the thiol groups present in the inner mitochondrial membrane [53,54,55]. In the present study, we observed that the genes involved in steroidogenesis, such as *Star*, *Hsd3b3*, and *Hsd17b1*, were significantly downregulated, which confirms the above hypothesis. Inhibition of the expression of genes associated with steroid biosynthesis or their activity was also demonstrated in our previous study on the testis of rats intravenously exposed to BSA-coated AgNPs [12]. Additionally, Lyu et al. [56] identified steroid hormone synthesis as one of the molecular pathways disturbed as a result of oral exposure to AgNPs. Furthermore, downregulation of the expression of *Star* gene, which encodes StAR protein that regulates the rate-limiting step in steroid biosynthesis, suggests that AgNPs impaired de novo hippocampal steroidogenesis.

Neurosteroids exert significant biological effects on the brain because they act as allosteric modulators of various neurotransmitters receptors, including gamma-aminobutyric acid A, N-methyl-D-aspartate, and serotonin receptors, and therefore regulate various brain functions such as cognition, locomotion, stress reactions, and anxiety [51]. In terms of cognitive skills, neurosteroids act as modulators of the activity and plasticity of neurons, synaptic conduction processes, neurogenesis, and learning as well as memory consolidation processes [57]. Oral exposure to AgNPs and Ag^+^ caused significant changes in the concentration of steroids in the hippocampus of rats, including, in particular, an increase in pregnenolone level in the AgNPs(PEG) group and a reduction in pregnenolone in the Ag^+^ group, as well as a reduction in progesterone and 17α-progesterone in the AgNPs (BSA), AgNPs (Cit), and Ag^+^ groups, an increase in 4-androsten-11β-ol-3.17-dione in AgNPs (BSA) and Ag^+^ groups, and an increase in DHT in AgNPs (BSA), AgNPs(Cit), and Ag^+^ groups. The behavioral test used to assess learning skills in these rats revealed impaired formation and consolidation of memory traces in the AgNPs(BSA)-treated group. The effects of this impairment were shown to be deficits of memory acquisition and maintenance of long-term memory [24]. Other authors also observed steroid-related behavioral disorders, including anxiety-like behavior in mice exposed to citrate-coated AgNPs [45].

The present study suggested that Ag^+^ and AgNPs differed in their mode of action on cells, as confirmed by the results of Fisher’s LDA. The Ag^+^ group was separated from both the control group and the groups that received AgNPs with different types of coating materials. This group was characterized by altered steroid synthesis and metabolism, which subsequently resulted in reduced levels of initial steroidogenesis metabolites, including progesterone and 17α-progesterone, and increased levels of highly potent steroid hormones that negatively affect the CNS (DHEA and DHEAS). Although decreased DHEA levels have been linked to several age-related diseases, in vitro and in vivo studies showed that this steroid can also exhibit neurotoxic effects at high concentrations [58,59]. Interestingly, a significant increase in the levels of DHEA and DHEAS was found only in the Ag^+^ group. These outcomes may result from AgNPs’ potential to induce mitochondrial damage, which other authors noted and described [53,54,55].

Interestingly, the outcomes of the present study revealed that surface functionalization is one of the most important factors influencing the biological effects of nanoparticles. The most significant changes observed in the present study include levels of oxidative stress and in the production of neurosteroids, with differences observed both in the concentration of neurosteroids and the expression of genes involved in their metabolism. These alterations were predominantly caused by BSA-coated AgNPs. The results of ANOVA and LDA showed that AgNPs(BSA) increased the activity of SOD and GPx and decreased the levels of the initial steroidogenesis metabolites (progesterone). The effect of AgNPs(BSA) was also indicated by decreased levels of androgen steroids, such as testosterone, and a simultaneous increase in the DHT concentration in the hippocampus, which suggests a probable reduction of testosterone levels by 5α-reductase in the peripheral tissues. On the other hand, no changes in the expression of *Srd5a1* gene, which encodes 5α-reductase, were observed in the hippocampus. In addition, only AgNPs(BSA) reduced the expression of the *Hsd17b10* gene that encodes 17β-hydroxysteroid dehydrogenase 10 (17β-HSD10) enzyme. Interestingly, 17β-HSD10 is a multifunctional mitochondrial enzyme that plays a key role in the metabolism and aging process of the CNS [60]. In the brain, this enzyme is involved in the conversion of androgen to estrogen, which further contributes to the pathology of neurodegenerative diseases by influencing memory mechanisms and mood control [61]. Importantly, reduced expression of *Hsd17b10* gene was noted in AgNPs(BSA) and Ag^+^ groups. Behavioral tests revealed that the animals of these two groups (AgNPs(BSA) and Ag^+^ groups) showed long-term memory and memory consolidation disorders, which are the characteristic symptoms of neurodegenerative diseases, such as Alzheimer’s disease. The 17β-HSD10 enzyme also contributes to the maintenance of mitochondrial integrity and is involved in the oxidation of fatty acids and steroids [62]. A reduction in the level of this enzyme was associated with enhanced oxidative stress, which is the characteristic effect of Ag^+^ ions and is responsible for mitochondrial dysfunction. Based on the experimental results, the toxic effects of AgNPs(BSA) can be attributed to the mechanism of Ag^+^ ions release, which is consistent with the results of previous studies. Besides the accumulation of a significant amount of Ag (determined by nanoSIMS) in the hippocampus of animals exposed to low doses of AgNPs, the presence of Ag^+^ ions (rather than nanoparticles) was also noted in our previous study [16]. 

The variation observed in the effects of AgNPs coated with different types of coating in the hippocampus can be related to the different degrees of stability provided by the different coating materials in the biological fluids, especially in the gastrointestinal tract [5,63]. BSA cannot form a permanent coating on AgNPs, as it is prone to digestion by the proteolytic enzymes in the gastrointestinal tract. After the digestion of BSA coating, AgNPs are surrounded by a protein corona formed by other available compounds, mainly food proteins [64]. The amount of Ag^+^ ions released into the surrounding environment is determined by the coating material used on AgNPs. 

The present study revealed only slight differences in the effects of AgNPs coated with PEG and citrate. AgNPs(Cit) induced higher levels of oxidative stress, as demonstrated by elevated levels of GPx and TBARS which may indicate that the oxidative stress-alleviating effect of antioxidant enzymes is increased, which further enhances lipid peroxidation. This condition can have serious implications for brain tissues, due to high amounts of unsaturated fatty acids which are highly susceptible to damage by ROS and the substantial toxicity of lipid peroxidation products on neurons.

Nevertheless, our previous results indicate that AgNPs(PEG) elicit a more robust systemic response by inducing systemic inflammation. In conclusion, the results of the present study confirmed the neurotoxic effects of AgNPs and showed that these effects could be minimized by modifying the physicochemical properties of nanoparticles, and the use of appropriate and coating is the most effective approach for this purpose.

## 4. Materials and Methods

### 4.1. Preparation and Characterization of Silver Nanoparticles

In the in vivo experiment, the studied animals were exposed to AgNPs with three different types of surface coating. AgNPs with bovine serum albumin (BSA; PlasmaChem, Berlin, Germany) coating and a nominal diameter of 20 ± 5 nm were prepared as described previously [65,66]. Briefly, 2 mg of AgNPs was dispersed in 800 μL of purified distilled water to form the nanoparticle stock solution. The stock solution was sonicated for 10 min on ice using a probe sonicator (Branson, Danbury, CT, USA) with 420 J m^−3^ total ultrasound energy. After sonication, 100 μL of 15% BSA and 100 μL of 10× phosphate-buffered saline (PBS) were added directly to the solution. AgNPs (nominal diameter of 25 nm) coated with sodium citrate were obtained from NanoCom-posix (San Diego, CA, USA). These nanoparticles had a hydrodynamic diameter of 25 nm and zeta potential (ζ) of −43 mV, according to the manufacturer’s information.

PEGylated AgNPs were prepared by mixing poly(ethylene glycol) methyl ether thiol (PEG, 5000 Da; Sigma-Aldrich, St. Louis, MO, USA) and 1 mg of AgNPs in 100 µL of aqueous SH-PEG solution (1 mg of PEG dissolved in 100 µL of water). Then, the reaction mixture was stirred for 2 h at room temperature. The zeta potential and hydrodynamic diameter of nanoparticles were determined by dynamic light scattering using the Zetasizer Nano ZS system (Malvern, UK) at 25 °C with a scattering angle of 173°. Stock solutions of PEG-coated AgNPs (pH 7.4) were diluted in water, and their zeta potentials were measured in triplicate with 14-sub runs, by applying the Smoluchowski limit for the Henry equation with a setting calculated for practical use (f(ka) = 1.5). In addition to zeta potential and hydrodynamic size, AgNPs with different types of coating (BSA, citrate, PEG) were evaluated for aggregation state and characterized by scanning electron microscopy (DSM 942, Carl Zeiss, Göttingen, Germany) as well as transmission electron microscopy (JOEL 1200 EX II, JOEL, Tokyo, Japan), and the results have been already published [3]. The characteristics of the AgNPs used in the in vivo experiment are described in Table 3.

### 4.2. Animals and Experimental Design

**Figure 5:**
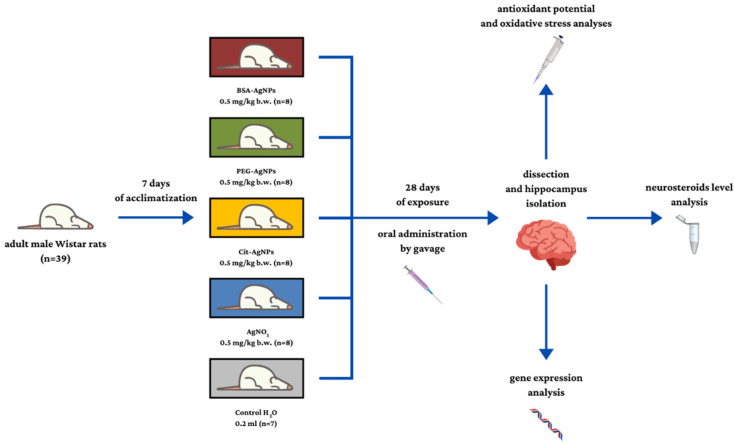
Scheme of the experimental design. Designed using elements by ©Canva via http://Canva.com (accessed on 23 December 2021, version used Canva 2.0).

The in vivo experiment was conducted on 10-week-old male Wistar rats (Wistar Cmbd:Wi strain) (n = 39), purchased from the Medical University of Bialystok, Center for Experimental Medicine (Polish Breeder’s register no. 003, GLP Certificate 17/2018/DPL). The animals were placed under standard housing conditions (12 h light/12 h dark cycles, temperature 22 °C, relative humidity 55%) and provided with both water and food ad libitum (Labofeed B maintenance diet, providing 67% of energy from carbohydrates, 8% from fat, and 25% from protein). After a 1-week adaptation period, the rats were assigned to one of the following groups: AgNPs(BSA) group (n = 8), AgNPs(PEG) group (n = 8), AgNPs(Cit) group (n = 8), Ag^+^ group (n = 8), or control (n = 7). The experimental animals received orally by gavage 0.5 mg/kg body weight (b.w.) of AgNPs or silver nitrate for 28 days, while the control group received 0.2 mL of H_2_O for 5 days a week. Changes in the weight of animals were monitored once a week throughout the experimental period. All the experimental procedures performed in the study were approved by the First Warsaw Local Ethics Committee for Animal Experimentation (application no. 788/2015, 25 May 2015) and carried out in accordance with the corresponding Polish legal regulations. The design of the experiment and description of the groups are provided in Figure 5.

### 4.3. Tissue Collection and Preparation

At the end of the experiment, the animals were sacrificed by isoflurane inhalation (Baxter Healthcare, Warsaw, Poland). The brains of the animals were excavated and rinsed in saline solution, and then the hippocampi were isolated from both hemispheres. The ventral parts were used for the analyses of total antioxidant status (TAS) and activities of glutathione peroxidase (GPx), glutathione reductase (GSR), and superoxide dismutase (SOD), as well as the level of lipid peroxidation (TBARS) and neurosteroids, while the dorsal parts were used for RNA isolation and gene expression analysis. Hippocampi were frozen in liquid nitrogen and stored at −80 °C for further biochemical analyses. To determine the antioxidant potential, the ventral parts were homogenized in PBS (pH 7.4; Sigma-Aldrich, St. Louis, MO, USA) in a tissue-to-buffer volume ratio of 1:5, using a homogenizer (Bio-Gen PRO 200, PRO Scientific, Oxford, MS, USA). The resulting homogenates were centrifuged for 5 min at 4 °C and 5000× *g* (Multifuge 3L-R centrifuge, Kendro, Hanau, Germany). After centrifugation, the supernatants were transferred to 200-µL test tubes and frozen at −80 °C for further analyses.

### 4.4. Neurosteroid Level in the Hippocampus

To determine the levels of neurotransmitters in the hippocampus (pregnenolone, progesterone, 17α-progesterone, allopregnanolone, dehydroepiandrosterone (DHEA), dehydroepiandrosterone sulfate (DHEAS), androstenedione, 17β-estradiol, testosterone, and dihydrotestosterone (DHT)), a quantitative analysis was carried out using quadrupole time-of-flight tandem mass spectrometry (SCIEX TripleTOF 5600+ DuoSpray Source for SCIEX TripleTOF 5600+ (TurboIonSpray and APCI) (Framingham, MA, USA). Identification of neurotransmitters was performed using the commercially available steroid standards (Sigma-Aldrich, St. Louis, MO, USA). Methanol and acetonitrile were HPLC/MS grade (JT Baker, Deventer, The Netherlands).To prepare the sample for the analysis, rat hippocampi were homogenized with 800 µL of acetonitrile and methanol mixture (1:1), and then vortexed (2000 rotations for 15 min) and centrifuged (13,000× *g* rpm for 15 min). After centrifugation, the supernatants were transferred to glass autosampler vials and placed in an autosampler at 4 °C. Chromatographic separation was performed using Hypersil chromatographic column (Phenomenex, Torrance, CA, USA), BDS C18, 150 × 4.6 mm, 5 mm with a Hypersil C18 guard column (10 × 2.1 mm, size 5 μm). The mobile phase used for the analysis consisted of methanol:formic acid (99:1, *v*/*v*) (A) and water:formic acid (99:1, *v*/*v*) (B), with the flow rate set constant at 500 µL min^−1^. The gradient elution program was as follows: starting with 100% A, 1.1–40 min linear gradient to 100% B, 40.1–55 min 100% B, and 55.1–60 min linear gradient to 100% A. The runtime was 60 min. The mass spectrometry parameters for optimized detection were as follows: curtain gas (N_2_) 25 psi, nebulizer gas (N_2_) 20 psi, heater gas (N_2_) 50 psi, ion source voltage floating 5500 V, and source temperature 500 °C. Samples with a heated electrospray ionization probe were measured in positive ionization mode. Every third sample analyzed using the Calibrant Delivery System (SCIEX) mass spectrometry system was autocalibrated using original calibrators (SCIEX). Method validation and quantitative analysis were carried out in the original SCIEX software (Analyst v1.7.1, PeakView v2.2, MasterView v1.3.1).

### 4.5. Antioxidant Potential and Oxidative Stress Analysis

The antioxidant potential of hippocampus homogenates was analyzed by measuring the activity of GPx, GSR, and SOD as well as TAS. The activity of enzymes was assessed spectrophotometrically using dedicated kits (Ransel, GR, and Ransod kits for GPx, GR, and SOD, respectively; Randox Laboratories, London, UK), following the manufacturer’s instructions, and light intensity was measured using Biochrom Anthos Zenyth 200 spectrophotometer (Cambridge, UK) at a wavelength of 505 nm. The results of the enzyme activity analysis were expressed in U/g of tissue. TAS was also assessed spectrophotometrically using a dedicated Total Antioxidant Status kit (Randox Laboratories, London, UK), as per the manufacturer’s instructions, and light intensity was measured using Biochrom Anthos Zenyth 200 spectrophotometer (Cambridge, UK) at a wavelength of 600 nm. The results of the TAS analysis were expressed in µmol/g of tissue. 

The level of lipid peroxidation was assessed as the concentration of TBARS and other secondary products of lipid peroxidation such as malondialdehyde (MDA) [67]. The hippocampus supernatants were mixed with 0.1 M sulfuric acid and 1% thiobarbituric acid (TBA) (Sigma-Aldrich, St. Louis, MO, USA). After warming at 90 °C for 1 h, the samples were cooled and mixed with n-butanol, and then the tubes containing samples were placed in ice until the two phases separated. The upper phase was isolated and incubated with TBA. Subsequently, the isolates were centrifuged for 5 min at 2000× *g*, and the absorbance was measured at 534 nm using Biochrom Anthos Zenyth 200 spectrophotometer (Cambridge, UK). TBARS concentration was calculated from a standard curve based on 1,1,3,3-tetramethoxypropane which converts to MDA upon hydrolysis during the assay, and the results were expressed as nmol MDA/mg tissue.

### 4.6. Gene Expression Analysis in Rat Hippocampus

Gene expression analysis was performed using the quantitative polymerase chain reaction (qPCR) method. RNA extraction from the rat hippocampus samples was carried out using RNeasy Lipid Tissue Mini Kit (Qiagen, Hilden, Germany), following the manufacturer’s instructions. The concentration and purity of RNA were determined using NanoDrop^TM^ 2000 spectrophotometer (Thermo Fisher Scientific, Waltham, MA, USA). Based on the absorbance ratios (A260/A280 and A260/A230), the samples were confirmed as pure and lacking protein residues. RNA integrity of several randomly selected samples was assessed using Agilent Bioanalyzer 2100 with an RNA 6000 Nano LabChip^®^ kit (Agilent Technologies, Palo Alto, CA, USA). The analysis showed a minimal degradation rate for RNA, with an RNA integrity number (RIN) of greater than 9. For PCR analysis, 1 µg of RNA was converted to its cDNA in a 20-µL reaction volume, using RT2 First Strand Kit (Qiagen, Hilden, Germany). Then, cDNA was diluted with 91 µL of ultraPure DNase/RNase-free distilled water and used for gene expression analysis which was carried out using RT2 Profiler PCR Arrays (Qiagen, Hilden, Germany), in accordance with the manufacturer’s instructions. RT2 Profiler PCR Arrays included primers for specific genes (*Ar*, cat. no. PPR44497A; *Cat*, cat. no. PPR42937A; *Cyp11a1*, cat. no. PPR42479A; *Cyp17a1*, cat. no. PPR44710A; *Cyp19a1*, cat. no. PPR47164A; *Cyp21a1*, cat. no. PPR66742A; *Esr1*, cat. no. PPR44939B; *Esr2*, cat. no. PPR48980A; *Gpx1*, cat. no. PPR45366A; *Gsr*, cat. no. PPR46891B; *Hsd3b*, cat. no. PPR48776C; *Hsd11b2*, cat. no. PPR44719A; *Hmox1*, cat. no. PPR57718A; *Hsd17b1*, cat. no. PPR44938B; *Hsd17b3*, cat. no. PPR45110A; *Hsd17b10*, cat. no PPR42824A; *Sod1*, cat. no. PPR43506A; *Sod2*, cat. no. PPR57578A; *Srd5a1*, cat. no. PPR43427F; *Star*, cat. no PPR45414A; *Sult2a2*, cat. no PPR75581A) (Qiagen, Hilden, Germany). The 25-µL reaction mixture used for PCR consisted of 1 µL of cDNA template, 11.5 µL of DNase/RNase-free distilled water, and 12.5 µL of RT2 SYBR Green/ROX qPCR Master Mix along with Hot-Start DNA Taq Polymerase, SYBR green, and ROX reference dye (Qiagen, Hilden, Germany). Amplification was performed in Stratagene Mx3005P qPCR thermocycler (Agilent Technologies, Palo Alto, CA, USA). After the first 10 min (95 °C), 40 consecutive cycles were performed, with each cycle involving 15-s reactions at 95 °C and 60-s reactions at 60 °C. Relative gene expression was calculated using the ΔΔCt method with phosphoglycerate kinase as the reference gene (*Pgk1*, cat. no. PPR56649C) (Qiagen, Hilden, Germany). The results were expressed as relative gene expression of the target gene vs. the reference genes (*Pgk1*), with the control group calculated as 1. 

### 4.7. Statistical Analysis

Data obtained from the in vivo experiment and biochemical assays were analyzed statistically using Statistica v. 13.3 PL software (StatSoft Polska Sp. z o.o., Kraków, Poland). Gene expression and antioxidant potential values were analyzed by one-way analysis of variance (ANOVA). Before each analysis, the assumptions of ANOVA were verified. The normality of residual distribution was tested using the Shapiro–Wilk test, and the equality of variance using the Brown–Forsythe test. Data that did not meet the assumption of normality were logarithmized. Differences between the groups were assessed using the Tukey post hoc test. The results were considered statistically significant at *p* < 0.05. GraphPad Prism (GraphPad Software 9.3.1) was used for creating all graphs. The experimental data from Fisher’s linear discriminant analysis (LDA) were also analyzed in R statistical software v. 3.3.3 (www.rproject.org/, accessed on 20 December 2021; R: The R Project for Statistical Computing). 

## 5. Conclusions

The present study revealed changes in the hippocampal concentrations of neurosteroids, as well as oxidative stress in the hippocampus, together with cognitive impairment, in rats exposed to silver. This may suggest that these mechanisms are responsible for the observed Ag neurotoxicity, which was strongly dependent on the coating material used on AgNPs and the Ag form. The study highlights that the effect of silver administered as AgNPs or as Ag^+^ varies from that of Ag^+^ released from AgNPs. Additionally, the results indicate that the mode of action of AgNPs with different types of coating is more similar to each other than to the action of Ag^+^ ions. Thus, it seems that the toxicity of AgNPs cannot be explained solely by the release of Ag^+^ ions.

## Figures and Tables

**Table 1:** Effect of oral administration of AgNPs with different types of coating (citrate, bovine serum albumin (BSA) and polyethylene glycol (PEG)) or Ag^+^: (0.5 mg/kg b.w.) on the relative expression of antioxidant defense genes vs. the reference phosphoglycerate kinase (*Pgk1*) gene.

Gene	Groups				
	AgNPs(BSA)	AgNPs(PEG)	AgNPs(Cit)	Ag^+^	Control
*Cat*	0.783 ± 0.054 ^a^	0.706 ± 0.041	0.818 ± 0.057	0.665 ± 0.039 ^a^	1.000 ± 0.009 ^b^
*Gsr*	0.819 ± 0.036	0.820 ± 0.054	0.890 ± 0.038	0.770 ± 0.028 ^a^	1.000 ± 0.092 ^b^
*Sod1*	0.997 ± 0.041	1.004 ± 0.031	1.012 ± 0.048	0.953 ± 0.011	1.000 ± 0.035
*Sod2*	0.793 ± 0.046	0.802 ± 0.013	0.894 ± 0.016	0.840 ± 0.013	1.000 ± 0.112
*Gpx1*	0.595 ± 0.054	0.553 ± 0.064	0.800 ± 0.174	0.550 ± 0.094	1.000 ± 0.362
*Hmox1*	0.895 ± 0.154	0.787 ± 0.125	0.823 ± 0.062	0.805 ± 0.110	1.000 ± 0.162

Data are presented in arbitrary units as a ratio of the expression of the target gene to the expression of the reference gene (*Pgk1*) with the control group calculated as 1. All values are expressed as mean ± SEM. ^a, b^ Statistically significant difference from the silver-exposed group according to the Tukey post hoc test (*p* < 0.05). The same letters indicate statistically significant results. *Cat*—catalase; *Gsr*—glutathione reductase; *Sod1*—superoxide dismutase 1; *Sod2*—superoxide dismutase 2; *Gpx1*—glutathione peroxidase 1; *Hmox1*—heme oxygenase 1.

**Table 2:** Effect of oral administration of AgNPs with different types of coating (citrate, bovine serum albumin (BSA), and polyethylene glycol (PEG)) or Ag^+^ (0.5 mg/kg b.w.) on the relative expression of the genes involved in neurosteroid synthesis and metabolism vs. the reference phosphoglycerate kinase (*Pgk1*) gene.

Gene	Groups				
	AgNPs (BSA)	AgNPs(PEG)	AgNPs(Cit)	Ag^+^	Control
*Cyp11a1*	0.804 ± 0.400	1.067 ± 0.577	0.740 ± 0.209	0.726 ± 0.130	1.000 ± 0.054
*Hsd17b3*	0.901 ± 0.143	0.883 ± 0.112	0.873 ± 0.068	0.671 ± 0.080	1.000 ± 0.067
*Srd5a1*	1.242 ± 0.067	1.006 ± 0.083	1.139 ± 0.059	1.141 ± 0.018	1.000 ± 0.065
*Sult2a2*	0.812 ± 0.256	0.871 ± 0.459	1.215 ± 1.069	0.689 ± 0.332	1.000 ± 0.235
*Cyp19a1*	0.595 ± 0.054	0.553 ± 0.064	0.800 ± 0.174	0.550 ± 0.094	1.000 ± 0.362

Data are presented in arbitrary units as a ratio of the expression of the target gene to the expression of the reference gene (*Pgk1*) with the control group calculated as 1. All values are presented as mean ± SEM. *Cyp11a1*—cytochrome P450, family 11, subfamily a, polypeptide 1; *Hsd17b3*—hydroxysteroid (17β) dehydrogenase 3; *Srd5a1*—steroid-5α-reductase, α-polypeptide 1 (3-oxo-5α-steroid delta 4-dehydrogenase α-1); *Sult2a2*—sulfotransferase family 2A, dehydroepiandrosterone (DHEA)-preferring, member 2; *Cyp19a1*—cytochrome P450, family 19, subfamily a, polypeptide 1.

**Table 3:** Characterization of AgNPs in water after dispersion (mean ± SD) (modified from Meczyńska-Wielgosz et al. [65].

	BSA-Coated AgNPs	PEG-Coated AgNPs	Citrate-Coated AgNPs
Nominal size of Ag particles [nm]	20 ± 5	25 ± 5	25 ± 5
Dynamic light scattering [nm]	84.4 ± 3.7	58.3 ± 6.5	27.5 ± 5.6
Polydispersity index	0.295	0.144 ± 0.06	0.308 ± 0.05
Zeta potential [mV]	−33.6	−30.2	−32.5

Data are expressed as mean ± SD (n = 3).

## Data Availability

The data that support the findings of this study are available on request from the corresponding author (K.D.).
